# Targeting IL-1β: a potential strategy for alleviating the immunopathology of aortic aneurysms

**DOI:** 10.3389/fimmu.2026.1821082

**Published:** 2026-05-28

**Authors:** Fengmei Zhang, Yuanpeng Liao, Di Zhao, Jiawei Guo

**Affiliations:** 1 Department of Cardiovascular Surgery, The First Affiliated Hospital of Yangtze University, Jingzhou, China; 2 Department of Pharmacology, School of Medicine, Yangtze University, Jingzhou, China

**Keywords:** aortic aneurysm, IL-1β, inflammation, macrophage polarization, targeted therapy

## Abstract

In recent years, the incidence of aortic aneurysms has been on the rise due to population aging, increased burden of chronic diseases, lifestyle changes, and advances in clinical technology. Identifying therapeutic targets for aortic aneurysms has become paramount in alleviating their immunopathology. Key pathological factors in aneurysm formation include inflammatory cell infiltration, metalloproteinase-mediated degradation of elastin and collagen, and increased proinflammatory factor activity. Macrophages play a pivotal role in vascular inflammation. Their polarization toward the M1 phenotype promotes IL-1β production. IL-1β contributes to aortic aneurysm inflammation by recruiting immune cells to the vascular wall, enhancing matrix metalloproteinase activation, and inducing apoptosis and phenotypic transformation of vascular smooth muscle cells. Thoracic and abdominal aortic aneurysms differ in terms of etiology, the cellular origin of IL-1β, and their degree of inflammation dependence, suggesting that IL-1β-related mechanisms are disease-specific. Current research indicates that inhibiting IL-1β activity and expression can alleviate aortic aneurysms. This review discusses the mechanisms by which IL-1β promotes aortic aneurysm inflammation and the principles underlying the alleviation of aortic aneurysm immunopathology.

## 1 Introduction

An aortic aneurysm is a pathological, permanent dilation of the aorta exceeding 50% of the normal vessel diameter. Primary risk factors include smoking, hypertension, atherosclerosis, and hyperlipidemia (1). Based on location, aortic aneurysms are classified as thoracic aortic aneurysms (TAAs), abdominal aortic aneurysms (AAAs), and transaortic aortic aneurysms (TAAAs) (2). TAAs can occur in the ascending aorta, the aortic arch, and the descending aorta, with the ascending aorta being the most common site (60%), followed by the descending aorta (40%) (3). AAAs commonly occur in the abdominal aortic segment below the renal arteries (4). Clinical data indicate that aortic aneurysms are the 15th leading cause of death in people aged 55 and older. In recent years, with advances in diagnostic technology and an aging population, the incidence of aortic aneurysms has been on the rise (5).Currently, the pathogenesis of aortic aneurysms remains unclear, with only a minority having identifiable causes. It is widely recognized that the development of aortic aneurysms is associated with adverse lifestyle habits such as smoking, hypertension, aging, hyperlipidemia, and other chronic diseases (6). Although these traditional risk factors are widely recognized, recent studies suggest that genetic susceptibility and epigenetic regulation may play a more significant role in the development of aortic aneurysms. This may explain the observed individual variations in the pathogenesis of aortic aneurysms.

TAAs and AAAs exhibit significant epidemiological heterogeneity. The incidence of TAAs is relatively low but on the rise, and the mortality rate following rupture is extremely high, reaching up to 80% (7). In contrast, the prevalence of AAAs in the global population aged 30 to 79 is 0.92%, with men having a significantly higher prevalence than women—approximately 3.7 times that of women; simultaneously, the mortality rate following AAA rupture is extremely high, with an overall mortality rate of approximately 81% (8). Regarding pathogenesis, TAAs are primarily attributed to genetic factors or connective tissue disorders (such as Marfan syndrome), with pathological features characterized by cystic necrosis of the aortic media and intrinsic apoptosis of smooth muscle cells, dominated by abnormalities in the TGF-β signaling pathway (9–11). In contrast, AAAs are strongly associated with smoking, atherosclerosis, and chronic inflammation. Pathologically, they show marked macrophage and T-cell infiltration in the adventitia and outer media. Excessive MMP-9 activation further contributes to extensive elastic fiber degradation. These conditions are often accompanied by mural thrombus formation and high expression of IL-1β mediated by the NLRP3 inflammasome (12–15).

Treatment for aortic aneurysms primarily relies on medication and surgical intervention. Although multiple drugs have been evaluated in animal models and clinical trials, none have been approved by the U.S. Food and Drug Administration (FDA) for treating aortic aneurysms due to the lack of confirmed efficacy in clinical studies (16). Currently, surgical intervention remains the mainstay of treatment for aortic aneurysms. Surgical intervention remains the primary means of preventing aortic aneurysm rupture and saving lives. It is indicated for female patients with aneurysms ≥5.0 cm in diameter and male patients with aneurysms ≥5.5 cm in diameter, while also considering other overall and specific patient characteristics (17–19). Surgical interventions primarily include open aortic repair (OAR) and endovascular aortic repair (EVAR). OAR involves thoracotomy or laparotomy to resect the diseased aortic segment and implant an artificial graft. This procedure is technically demanding, requires high proficiency, and carries a higher rate of postoperative complications. Elderly patients or those with concomitant cardiac, pulmonary, hepatic, or renal insufficiency, or poor systemic condition often cannot tolerate the risks of open surgery (20). EVAR offers less trauma and simpler operation, but it cannot address complex aortic aneurysms due to insufficient proximal anchoring and issues involving visceral branch arteries (21). Therefore, treatment strategies for aortic aneurysms still have significant room for improvement. On one hand, drug therapies have limited and unclear efficacy; on the other, surgical interventions have a narrower scope of application. This underscores the urgency of developing targeted drugs capable of slowing aortic aneurysm progression.

The formation of aortic aneurysms is a complex process involving multiple cellular and molecular mechanisms. Key pathological factors in aneurysm development include inflammatory cell infiltration of the vascular wall, matrix metalloproteinase (MMP) degradation of elastin and collagen, loss of vascular smooth muscle cells (VSMCs), increased proinflammatory cytokine activity, exacerbated oxidative tissue injury, and neovascularization (22). Among these, the inflammatory response represents a central component of the aortic aneurysm pathology. It manifests as extensive infiltration of macrophages, neutrophils, and T lymphocytes into the vascular wall, accompanied by the secretion of chemokines, cytokines, and reactive oxygen species (ROS). This creates a persistent inflammatory microenvironment within the vessel wall, thereby exacerbating vascular damage (22). Notably, inflammation plays a dual role in aortic aneurysms. During the early stages of aneurysm formation, an appropriate inflammatory response serves as a reparative reaction to injury. However, under the persistent influence of risk factors, dysregulation of inflammation shifts the response toward tissue destruction. Future therapeutic approaches for aortic aneurysms must involve multi-targeted interventions addressing inflammation, matrix degradation, and vascular remodeling, rather than relying solely on inflammatory modulation.

Interleukin-1β (IL-1β), a member of the cytokine family, plays a pivotal role in initiating and amplifying inflammatory responses (23). IL-1β primarily originates from activated immune cells such as macrophages, monocytes, and dendritic cells (24). Additionally, B lymphocytes and natural killer cells can also produce IL-1β (25).Research indicates that IL-1β primarily upregulates the expression of vascular endothelial adhesion molecules (VCAM-1, ICAM-1), chemotactic molecules (MCP-1, IL-8), and proinflammatory factors through key signaling pathways such as NF-κB. This recruitment of inflammatory cells leads to the formation of a self-sustaining inflammatory microenvironment within the vascular wall (26). Within this cycle, pathological processes—including inflammatory cell infiltration, MMPs-mediated extracellular matrix (ECM) degradation, vascular smooth muscle cell apoptosis and transdifferentiation, and endothelial dysfunction—mutually reinforce each other, collectively accelerating aortic aneurysm progression (27, 28). Given IL-1β’s central role in aortic aneurysms, it has emerged as a focal point in research and therapeutic strategies for treating these conditions. TAAs and AAAs differ in etiology, cellular composition, pathological features, and dependence on inflammation. IL-1β plays a key role in inflammatory amplification, vascular wall disruption, and aneurysm progression. Therefore, this review discusses the activation mechanisms, pathological effects, targeted therapeutic strategies, and clinical translation prospects of IL-1β in different types of aortic aneurysms. It covers both TAAs and AAAs, but places particular emphasis on the IL-1β-related inflammatory axis.

Unlike previous reviews that mainly focused on the inflammatory microenvironment of aortic aneurysms or individual therapeutic strategies, this review centers on IL-1β. It systematically summarizes its upstream activation mechanisms, cellular origins, and disease-specific roles in TAAs and AAAs. It also discusses its interactions with NETs, IL-6, and TGF-β pathways, as well as therapeutic advances targeting the IL-1β/NLRP3 axis. The innovation of this paper lies in constructing a comprehensive framework for the role of IL-1β in the pathogenesis and progression of aortic aneurysms from the perspective of “common inflammatory mechanisms—disease specificity—translation to precision-targeted therapy”.

## 2 Activation and production of IL-1β in aortic aneurysms

**Figure 1:**
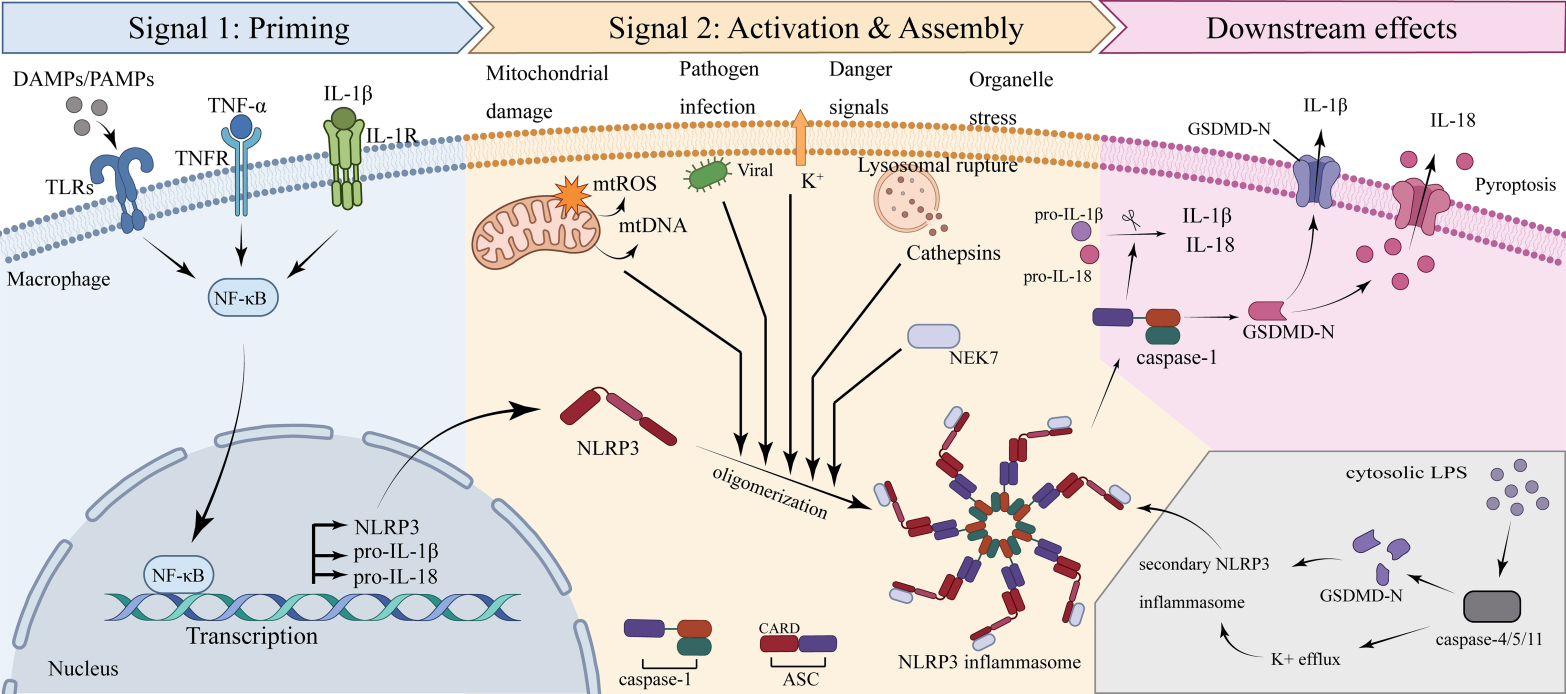
NLRP3 inflammasome activation and IL-1β maturation in aortic aneurysms. Danger signals activate the NLRP3 inflammasome through a two-signal mechanism. Priming signals induce the transcription of NLRP3, pro-IL-1β, and pro-IL-18 via NF-κB, whereas activation signals promote inflammasome assembly and caspase-1 activation. Caspase-1 cleaves pro-IL-1β, pro-IL-18, and GSDMD, leading to IL-1β/IL-18 release, pyroptosis, and vascular inflammation. ASC, apoptosis-associated speck-like protein containing a CARD; CARD, caspase recruitment domain; DAMPs, damage-associated molecular patterns; GSDMD, gasdermin D; IL, interleukin; LPS, lipopolysaccharide; mtDNA, mitochondrial DNA; mtROS, mitochondrial reactive oxygen species; NEK7, NIMA-related kinase 7; NF-κB, nuclear factor-κB; NLRP3, NOD-like receptor family pyrin domain-containing 3; PAMPs, pathogen-associated molecular patterns; TLRs, Toll-like receptors; TNF-α, tumor necrosis factor-α; TNFR, tumor necrosis factor receptor.

In aortic aneurysms, IL-1β production represents a complex, multi-step biological process involving multiple cell types (**Figure 1**). It begins with the synthesis of inactive pro-IL-1β, triggered by danger signals. This process is then integrated and amplified through pathways centered on the NLRP3 inflammasome, ultimately leading to massive IL-1β release (29). From a cellular perspective, IL-1β production also relies on both vascular-intrinsic cells and locally infiltrating immune cells (30). Under specific pathological conditions, these cells collectively form an IL-1β cellular source network through distinct molecular mechanisms, driving the development and progression of aortic aneurysms.

### 2.1 Danger signals and NLRP3 inflammasome

The NLRP3 inflammasome is a characteristic inflammatory multiprotein complex within the NOD-like receptor (NLR) family, composed of the NLRP3 receptor protein, ASC adaptor protein, and caspase-1 precursor. These three components form a functional complex through domain-mediated interactions (31).

The NLRP3 inflammasome is primarily expressed in myeloid innate immune cells, but under specific conditions it can also be activated in a variety of other cell types (32).The macrophages infiltrating the aortic wall are the primary site for NLRP3 activation and IL-1β production (14). As an intracellular pattern recognition receptor, NLRP3 detects various damage-associated molecular patterns (DAMPs) and pathogen-associated molecular patterns (PAMPs), and forms the NLRP3 inflammasome complex by recruiting ASC and pro-caspase-1 (33). DAMPs and PAMPs activate TLRs, which subsequently activate the NF-κB pathway via adaptor proteins such as MyD88 and TRIF, thereby inducing the expression of NLRP3 and pro-IL-1β/pro-IL-18 (34). It is worth noting that TLRs are not direct receptors or core regulators of IL-1β signaling; their primary role is to induce pro-IL-1β expression via the NF-κB pathway (35). The classical activation of the NLRP3 inflammasome typically follows a two-signal mechanism. Signal 1 is mainly triggered by PAMPs, DAMPs, and inflammatory stimuli such as TNF-α and IL-1β. These signals activate NF-κB through receptors including TLRs, IL-1R, and TNFR. Activated NF-κB translocates into the nucleus and upregulates NLRP3, pro-IL-1β, and pro-IL-18 expression, thereby preparing the molecular basis for inflammasome assembly (36). Signal 2 is induced by various cellular danger signals, including K^+^ efflux, mitochondrial damage and mtROS/mtDNA release, lysosomal rupture and cathepsin release, organelle stress, and pathogen infection (37). These signals promote the binding of NLRP3 to NEK7 and subsequent oligomerization (38). Subsequently, ASC is recruited by NLRP3 via PYD-PYD interactions and forms filamentous oligomers; it then recruits pro-caspase-1 via CARD-CARD interactions and promotes its self-cleavage to activate caspase-1 (39). Activated caspase-1 cleaves pro-IL-1β and pro-IL-18 to generate active IL-1β and IL-18, while also cleaving Gasdermin-D. The N-terminal region of Gasdermin-D forms pores in the cell membrane, promoting the release of IL-1β and IL-18 and triggering a form of programmed inflammatory cell death known as pyroptosis, which further exacerbates vascular inflammation in aortic aneurysms (40). Additionally, cytoplasmic LPS can activate caspases-4/5/11 via non-classical pathways, inducing GSDMD cleavage and K^+^ efflux, which in turn promotes the activation of the NLRP3 inflammasome (41).

In AAA, IL-1β activation is more frequently associated with atherosclerosis, lipid peroxides, cholesterol crystals, chronic inflammatory cell infiltration, and thrombus-related inflammation; whereas in TAA, IL-1β activation must be understood within the context of medial degeneration, VSMC stress, genetic/signaling pathway abnormalities, and intimal inflammation associated with the dissection (9, 42).

### 2.2 Immune cells and vascular innate cells

In terms of differences between TAAs and AAAs, the sources of IL-1β in AAAs tend to be predominantly infiltrating macrophages, monocytes, and neutrophils; whereas in TAAs, IL-1β primarily originates from VSMCs and T/B cells (43).

From the perspective of cell-cell interactions, IL-1β production is not generated independently by a single cell type but is regulated by a dynamic network formed by vascular endothelial cells and infiltrating immune cells. Mechanical stress, ROS, and DAMPs first induce stress responses in endothelial cells and VSMCs. These cells express adhesion molecules and chemokines, thereby promoting the recruitment of monocytes and neutrophils to the vascular wall. Subsequently, macrophages become a major site of NLRP3 inflammasome activation and IL-1β maturation (6, 14). The released IL-1β then acts via paracrine signaling on endothelial cells and VSMCs, further inducing endothelial dysfunction, VSMC phenotypic transformation, MMP expression, and ECM degradation (44). Therefore, IL-1β signaling in aortic aneurysms manifests as a bidirectional amplification process between vascular endothelial cells and immune cells, rather than a linear response originating from a single cell source.

Macrophages have been demonstrated as a key component in the aortic aneurysm inflammatory process and a primary source of IL-1β (45). Within aortic aneurysms, macrophages exhibit a significant M1/M2 polarization imbalance, characterized by the absolute predominance of persistently M1-polarized macrophages (46). This imbalance leads to massive release of proinflammatory cytokines such as IL-1β and TNF-γ, along with extracellular matrix degradation, perpetuating chronic inflammation in aortic aneurysms (47). Studies indicate that in human AAA specimens and mouse models, M1 markers (e.g., iNOS, IL-1β) are significantly elevated, while M2 markers (e.g., CD206, Arg-1) show minimal increase; Inhibiting macrophage polarization toward the M1 phenotype significantly attenuates extracellular matrix degradation and smooth muscle cell apoptosis in arterial walls, indicating the potential regulatory role of M1 macrophages in IL-1β-driven inflammatory cascades (47). Recruited monocytes mainly differentiate into classically activated M1 macrophages. These cells release IL-1β, IL-6, TNF-α, and other pro-inflammatory cytokines, thereby amplifying local inflammation (45, 48).

Neutrophils, meanwhile, can provide inflammasome activation signals through pathways such as NETosis and are themselves capable of producing IL-1β (49).

VSMCs and other vascular-intrinsic cells produce IL-1β via the NLRP3 inflammasome pathway (50, 51). In aortic aneurysms, VSMCs may act as “sensors” of local mechanical stress and biochemical danger signals. These stimuli can trigger NLRP3 inflammasome assembly in VSMCs. VSMCs may also serve as a “platform” for caspase-1 recruitment and activation. Activated caspase-1 cleaves pro-IL-1β into mature IL-1β, thereby promoting inflammation and matrix degradation (52).

Other cells such as dendritic cells, B lymphocytes, and NK cells also possess the capacity to produce IL-1β under certain conditions, collectively forming a multicellular cooperative inflammatory network (25).

### 2.3 Biological functions of IL-1β

IL-1β is a key proinflammatory cytokine released by innate immune cells upon recognizing pathogens or danger signals through pattern recognition receptors, activating critical pathways such as NLRP3 inflammasome activation (53). On one hand, IL-1β promotes C-reactive protein (CRP) expression to limit pathogen spread (54, 55). Simultaneously, as a central mediator of inflammation, IL-1β significantly upregulates the expression of adhesion molecules and chemokines in vascular smooth muscle cells (VSMCs), promoting the recruitment and infiltration of additional immune cells to the inflammatory site (56). Furthermore, IL-1β acts on VSMCs to promote their transformation and apoptosis, impairing their ability to maintain vascular tone and secrete extracellular matrix (57). Furthermore, IL-1β upregulates the expression of multiple proteolytic enzymes, promoting extracellular matrix degradation and leading to vascular wall structural disruption and dilation (58). In inflammatory diseases, IL-1β directly participates in and amplifies local and systemic inflammatory responses, enhancing the body’s defense against pathogens (59). IL-1β also promotes apoptosis through receptor-mediated signaling. It upregulates pro-apoptotic proteins, induces mitochondrial dysfunction, and activates apoptotic effectors such as caspase-3, caspase-8, and caspase-9. This disrupts the balance between cell survival and death, ultimately promoting apoptosis (60, 61).

In addition, IL-1β bridges the innate and adaptive immune systems. By promoting the activation of T cells and B cells, it enhances the body’s specific immune response and plays a crucial role in eliminating pathogens and damaged cells (62). However, IL-1β’s actions require precise regulation: binding to IL-1 receptors triggers downstream signaling. Excessive production or overly potent signaling may activate the NF-κB pathway, releasing massive proinflammatory factors and triggering life-threatening “cytokine storms” that cause systemic tissue damage. Persistent abnormal IL-1β activation contributes to the development of autoimmune or inflammatory diseases and chronic inflammation (63, 64). Notably, sustained excessive IL-1β activation is a core pathological factor causing abnormal aortic dilation and disrupting vascular homeostasis. Suppressing IL-1β production through knockout or pharmacological inhibition significantly reduces aortic dilation and improves vascular structure (65). Thus, IL-1β exhibits dual roles in immunoregulation and pathological processes: it serves as both a necessary defense and repair factor and an important potential therapeutic target.

## 3 Pathophysiological characteristics of aortic aneurysms

### 3.1 Pathological features of aortic aneurysms

The complex pathophysiological mechanisms of aortic aneurysms remain incompletely understood. The core pathological mechanism involves progressive destruction and remodeling of the aortic wall. This process is characterized by wall dilatation and thinning, elastic fiber rupture and degradation, vascular smooth muscle cells(VSMC) apoptosis and phenotypic transformation, and an imbalance between extracellular matrix(ECM) synthesis and degradation (9).

Histologically, aortic walls in aneurysm patients exhibit marked inflammatory cell infiltration, predominantly macrophages followed by neutrophils, T lymphocytes, and B lymphocytes (66). These inflammatory cells concentrate primarily in the media and adventitia, with partial infiltration into the intima (66). Macrophages participate in vascular wall inflammation and tissue remodeling by secreting multiple pro-inflammatory cytokines (e.g., IL-1β, TNF-α, IL-6), MMPs, and ROS (67).

VSMCs are the primary functional cells in the aortic wall, playing a role in maintaining vascular tone and structural stability during pathophysiological processes (68). In the pathological process of aortic aneurysms, VSMCs undergo phenotypic transformation from contractile to synthetic, losing their contractile function while gaining enhanced proliferation and migration capabilities (69). Additionally, massive VSMC apoptosis occurs, leading to thinning of the vascular wall’s media and disruption of structural integrity (70). Research indicates that multiple factors, including inflammatory mediators, oxidative stress, and mechanical stress, can induce VSMC apoptosis and phenotypic transformation (71).

The extracellular matrix (ECM), a critical component of the aortic wall comprising collagen, elastic fibers, and proteoglycans, relies on a dynamic balance between synthesis and degradation to maintain vascular wall structure and function (72). During aortic aneurysm pathogenesis, MMP expression and activity significantly increase. These enzymes degrade collagen and elastic fibers, leading to substantial ECM loss (73). Concurrently, insufficient collagen synthesis or abnormal deposition also compromises vascular wall elasticity and strength, exacerbating aortic aneurysm dilation (74).

The destruction of elastic fibers represents one of the most characteristic pathological alterations in aortic aneurysms (75). Composed of elastin and microfilaments, elastic fibers constitute the key structural elements maintaining the elasticity and recoil function of the aorta (76). In the aortas of patients with aortic aneurysms, elastic fiber rupture, fragmentation, and degradation occur, resulting in diminished vascular elasticity. This inability to effectively withstand blood flow pressure subsequently leads to progressive aneurysmal expansion (77). Research indicates that matrix metalloproteinases (MMPs) and elastase are primary enzymes responsible for elastic fiber degradation (78).

### 3.2 Risk factors associated with aortic aneurysm development

The development of aortic aneurysms results from the combined effects of genetic and environmental factors. On the basis of damage caused by genetic defects, a series of acquired risk factors exert long-term, cumulative effects, ultimately leading to an imbalance in vascular wall homeostasis (79). Genetically, clinical data indicate that familial aortic aneurysms exhibit significantly higher incidence than sporadic cases (80). Multiple susceptibility genes play crucial roles in the hereditary transmission of aortic aneurysms. Mutations in genes associated with the transforming growth factor beta (TGF-β) signaling pathway (e.g., TGFBR1, TGFBR2, SMAD3) constitute the core genetic defect underlying familial thoracic aortic aneurysms (81). These mutations severely disrupt the proliferation, differentiation, and normal synthesis and metabolism of extracellular matrix in VSMCs, fundamentally compromising the structural integrity of the aortic wall (82). Additionally, polymorphisms in MMPs and collagen genes (e.g., COL3A1) influence the degradation-repair balance of major vascular wall components from different angles, increasing genetic susceptibility to the disease (83, 84).

Meanwhile, multiple environmental and acquired factors play a crucial “catalytic” and “promoting” role in the progression of aortic aneurysm disease. Age is an unavoidable risk factor for aortic aneurysms. With aging, the vascular wall undergoes degenerative changes, including elastic fiber degradation, reduced VSMC number and function, and impaired repair capacity. These changes collectively increase susceptibility to aneurysm formation (85, 86). Among numerous modifiable factors, smoking poses the most prominent threat to the aortic wall (87). Harmful substances in tobacco inflict multiple direct damages to the aortic wall by inducing persistent multifaceted inflammation, exacerbating oxidative stress, inhibiting collagen synthesis, and abnormally elevating MMP activity (88). Chronic hypertension subjects vascular walls to persistently elevated mechanical stress, causing endothelial injury and activating the harmful renin-angiotensin-aldosterone system, thereby accelerating pathological vascular remodeling (89). Atherosclerosis, particularly closely linked to abdominal aortic aneurysms, contributes significantly to aneurysm formation and progression through chronic inflammation and internal damage associated with plaque development (27, 90). Furthermore, existing research indicates that metabolic disorders such as obesity, diabetes, and hyperlipidemia synergistically amplify aortic wall injury risks by promoting atherosclerosis, exacerbating endothelial dysfunction, and intensifying chronic inflammatory responses. This holds significant implications for the pathogenesis of vascular diseases like aortic aneurysms (91, 92).

## 4 Core mechanisms of IL-1β in aortic aneurysm development

### 4.1 IL-1β drives the inflammatory cascade in the vascular wall

**Figure 2:**
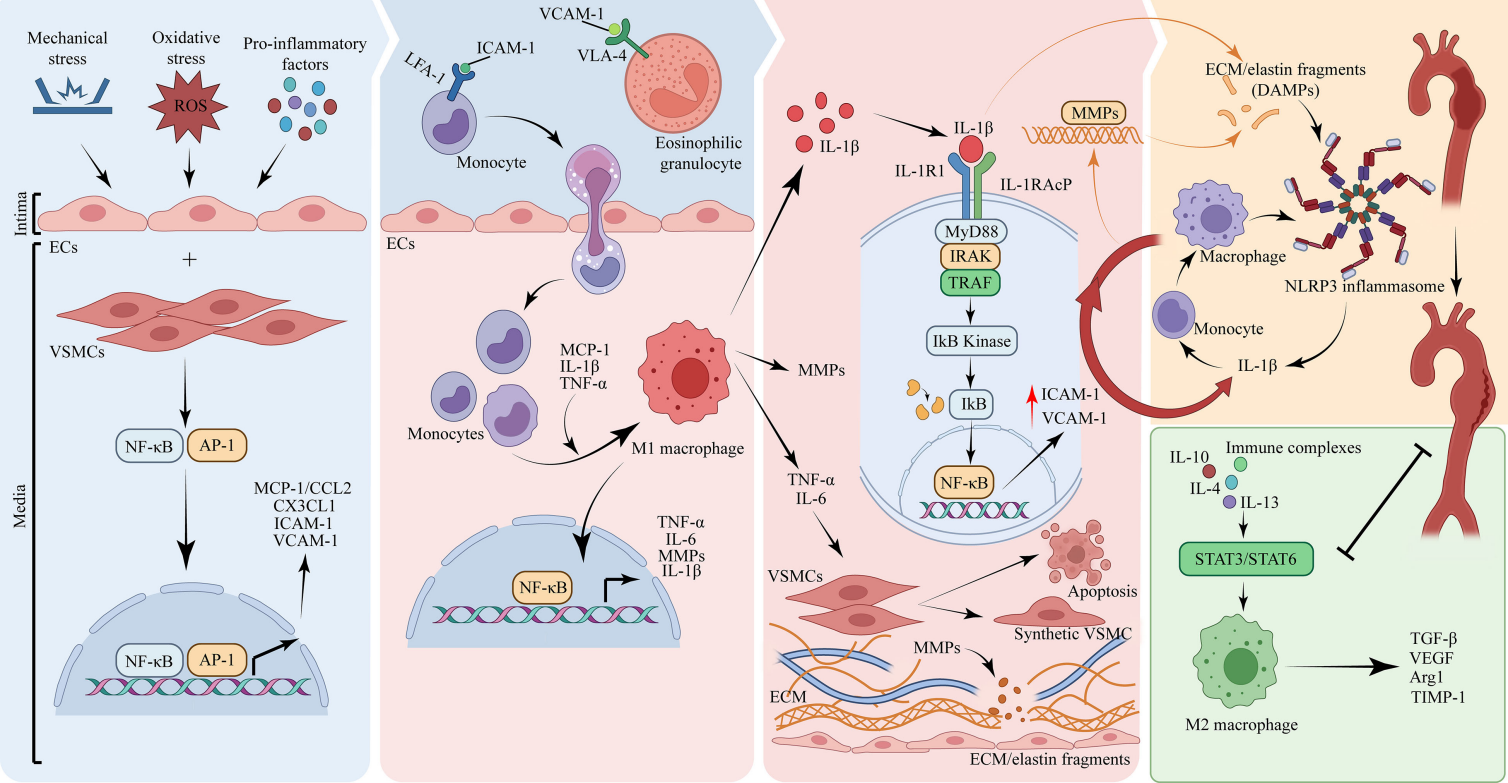
IL-1β-driven inflammatory amplification in aortic aneurysm progression. Mechanical stress, oxidative stress, and inflammatory stimuli activate endothelial cells and VSMCs, promoting adhesion molecule and chemokine expression. Recruited monocytes differentiate into macrophages, particularly M1 macrophages, which secrete IL-1β, TNF-α, IL-6, and MMPs. IL-1β further enhances leukocyte recruitment, macrophage activation, and ECM degradation, forming a self-sustaining inflammatory loop. AP-1, activator protein-1; Arg-1, arginase-1; CCL2, C-C motif chemokine ligand 2; CX3CL1, C-X3-C motif chemokine ligand 1; DAMPs, damage-associated molecular patterns; ECs, endothelial cells; ECM, extracellular matrix; ICAM-1, intercellular adhesion molecule-1; IL, interleukin; IL-1R1, interleukin-1 receptor type 1; IL-1RAcP, interleukin-1 receptor accessory protein; IRAK, interleukin-1 receptor-associated kinase; LFA-1, lymphocyte function-associated antigen-1; MCP-1, monocyte chemoattractant protein-1; MMPs, matrix metalloproteinases; MyD88, myeloid differentiation primary response 88; NF-κB, nuclear factor-κB; STAT, signal transducer and activator of transcription; TGF-β, transforming growth factor-β; TIMP-1, tissue inhibitor of metalloproteinase-1; TRAF, tumor necrosis factor receptor-associated factor; VCAM-1, vascular cell adhesion molecule-1; VEGF, vascular endothelial growth factor; VLA-4, very late antigen-4; VSMCs, vascular smooth muscle cells.

During aortic aneurysm pathogenesis, the inflammatory cascade exhibits a self-propagating, progressively amplified vicious cycle. IL-1β plays a central driving role by establishing and sustaining this self-reinforcing inflammation-destruction cycle (**Figure 2**). Chronologically, this process can be broadly divided into early endothelial cell stress, followed by immune cell recruitment and activation; mid-stage IL-1β-mediated inflammatory amplification; and late-stage reactivation driven by ECM degradation and DAMPs release. However, this inflammatory cascade is more pronounced and widespread in AAAs due to their etiology; in contrast, in TAAs—particularly those of hereditary or ascending aortic origin—dysfunction of vascular smooth muscle cells (VSMCs), disruption of ECM homeostasis, degeneration of elastic fibers, and abnormal TGF-β signaling constitute the key early and core pathological foundations (93).

In the early stages of aortic aneurysm formation, mechanical stress, oxidative stress, and inflammatory stimuli act on vascular intrinsic cells, particularly endothelial cells and VSMCs. These stimuli activate two key transcription factor pathways: NF-κB and AP-1 (94). Activation of these pathways induces vascular smooth muscle cells and endothelial cells to express large quantities of chemokines (e.g., MCP-1/CCL-2, CX3CL1) and adhesion molecules (e.g., ICAM-1, VCAM-1) (95). Upregulated expression of adhesion and chemotactic molecules in aortic aneurysms collectively promotes monocyte and macrophage infiltration into blood vessels, playing a central role in the inflammatory response (96).

Research indicates that ICAM-1 (intercellular adhesion molecule-1) primarily binds to LFA-1, an integrin widely expressed on leukocyte surfaces, participating in the migration and adhesion of nearly all leukocyte types; while VCAM-1 (vascular cell adhesion molecule-1) primarily binds to integrin VLA-4 on specific leukocyte subsets (lymphocytes, eosinophils, and basophils), facilitating their adhesion and migration (97). ICAM-1 and VCAM-1 are cell surface adhesion molecules belonging to the immunoglobulin superfamily, primarily expressed on vascular endothelial cells (98). Under normal conditions, they are generally not expressed or are lowly expressed; however, their expression is upregulated upon stimulation by inflammatory signals, thereby mediating leukocyte adhesion and migration (99, 100).

Subsequently, monocytes that have entered the vascular wall differentiate into macrophages under the influence of the local microenvironment (e.g., high levels of MCP-1, IL-1β, and TNF-α) (101). During persistent inflammation, NF-κB and AP-1 promote macrophage polarization toward the M1 phenotype. M1 macrophages express high levels of pro-inflammatory mediators, including iNOS, IL-12, and IL-1β, which further amplify inflammation (102). Once the inflammation amplification phase begins, IL-1β secreted by M1 macrophages can further promote the upregulation of adhesion molecules and chemokines in endothelial cells and VSMCs, recruiting more immune cells and forming a positive feedback loop (57).

IL-1β upregulates the expression of adhesion molecules through specific molecular mechanisms. It is one of the key inflammatory cytokines involved in ICAM-1 and VCAM-1 expression (103). First, IL-1β binds to IL-1R1 on the surface of vascular endothelial cells, subsequently forming a heterodimeric complex with the IL-1 receptor accessory protein (IL-1RAcP). This activates the downstream MyD88/IRAK/TRAF signaling pathway, which in turn activates IκB kinase. This leads to the phosphorylation and degradation of IκB protein, releasing NF-κB into the nucleus (57). Nuclear NF-κB binds to κB elements on the ICAM-1 and VCAM-1 gene promoters, promoting transcription of ICAM-1 and VCAM-1, ultimately leading to the expression of these adhesion molecules on the surface of vascular endothelial cells (104). Studies have demonstrated that IL-1β significantly upregulates ICAM-1 and VCAM-1 expression in human aortic endothelial cells (hAECs) cultured *in vitro* (105).

Concurrently, M1-polarized macrophages secrete cytokines including MCP-1/CCL-2, IL-6, TNF-α, and MMPs. Among these, TNF-α and IL-6 promote VSMC transformation or apoptosis, while MMPs degrade elastic fibers and collagen, compromising aortic wall structural integrity and accelerating arterial dilation (106–108). As the disease progresses to the stage of structural damage, extracellular matrix fragments generated by MMP degradation—such as elastin fragments—can serve as potent injury-associated molecular patterns (109). These DAMPs are recognized by pattern recognition receptors on immune cells like macrophages, further activating NLRP3 inflammasomes and leading to increased maturation and release of IL-1β (110). IL-1β secreted by M1 macrophages not only promotes endothelial cell expression of adhesion molecules. but also maintain and exacerbate the M1 polarization of macrophages themselves through autocrine and paracrine mechanisms. This creates a closed-loop inflammatory cycle where structural damage itself drives a new round of inflammation (111).

M2 macrophages are pivotal in regulating the transition from the inflammatory to the reparative phase of aortic aneurysm pathology. Theoretically, the body automatically initiates endogenous repair programs during inflammation or chronic disease. However, in the development of aortic aneurysms, the generation of M2 macrophages associated with endogenous repair is suppressed, impairing tissue repair and remodeling functions in the aortic wall (52). Within the aortic aneurysm microenvironment, signaling molecules such as IL-4, IL-10, IL-13, or immune complexes can induce macrophage polarization toward the M2 phenotype by activating downstream transcription factors like STAT3 and STAT6 (45, 112). This process is accompanied by the secretion of characteristic markers including TGF-β, VEGF, arginase-1 (Arg-1), and TIMP-1 (96).Among these, Arg-1 competes with inducible nitric oxide synthase (iNOS) for the substrate L-arginine, depleting L-arginine and thereby inhibiting nitric oxide (NO) production to reduce oxidative stress. TGF-β and IL-10 exert anti-inflammatory and tissue-repairing effects, jointly mediating vascular remodeling and immune suppression (113, 114). Consequently, within the aortic aneurysm microenvironment, pro-inflammatory M1 macrophages and the key pro-inflammatory cytokine IL-1β persistently dominate, while reparative M2 macrophages remain relatively deficient. This imbalance impairs tissue repair and vascular remodeling functions in the aortic wall, leading to escalating inflammation and structural deterioration. This severe disruption of the pro-inflammatory/repair balance eliminates intrinsic counterbalancing mechanisms for IL-1β-driven inflammation and destructive cycles, perpetuating a vicious cycle.

The inflammatory vicious cycle can be described as a time-dependent process. In the early stage, mechanical stress, ROS, and inflammatory stimuli activate endothelial cells and VSMCs, leading to the upregulation of ICAM-1, VCAM-1, MCP-1, and other adhesion or chemotactic molecules. Monocytes and other immune cells are then recruited to the vascular wall and differentiate into M1 macrophages. During the inflammatory amplification phase, M1 macrophages release IL-1β, MMPs, and other pro-inflammatory mediators. IL-1β further acts on endothelial cells and VSMCs, enhancing the expression of adhesion molecules, chemokines, and proteolytic enzymes. In the structural destruction phase, ECM degradation and elastic fiber rupture release additional DAMPs, which reactivate the NLRP3 inflammasome and promote further IL-1β maturation and release. Concurrently, the generation of M2 macrophages is suppressed, disrupting the balance between destruction and repair, and ultimately driving the progression and rupture of the aortic aneurysm.

### 4.2 IL-1β disrupts vascular wall homeostasis and structural integrity

**Figure 3:**
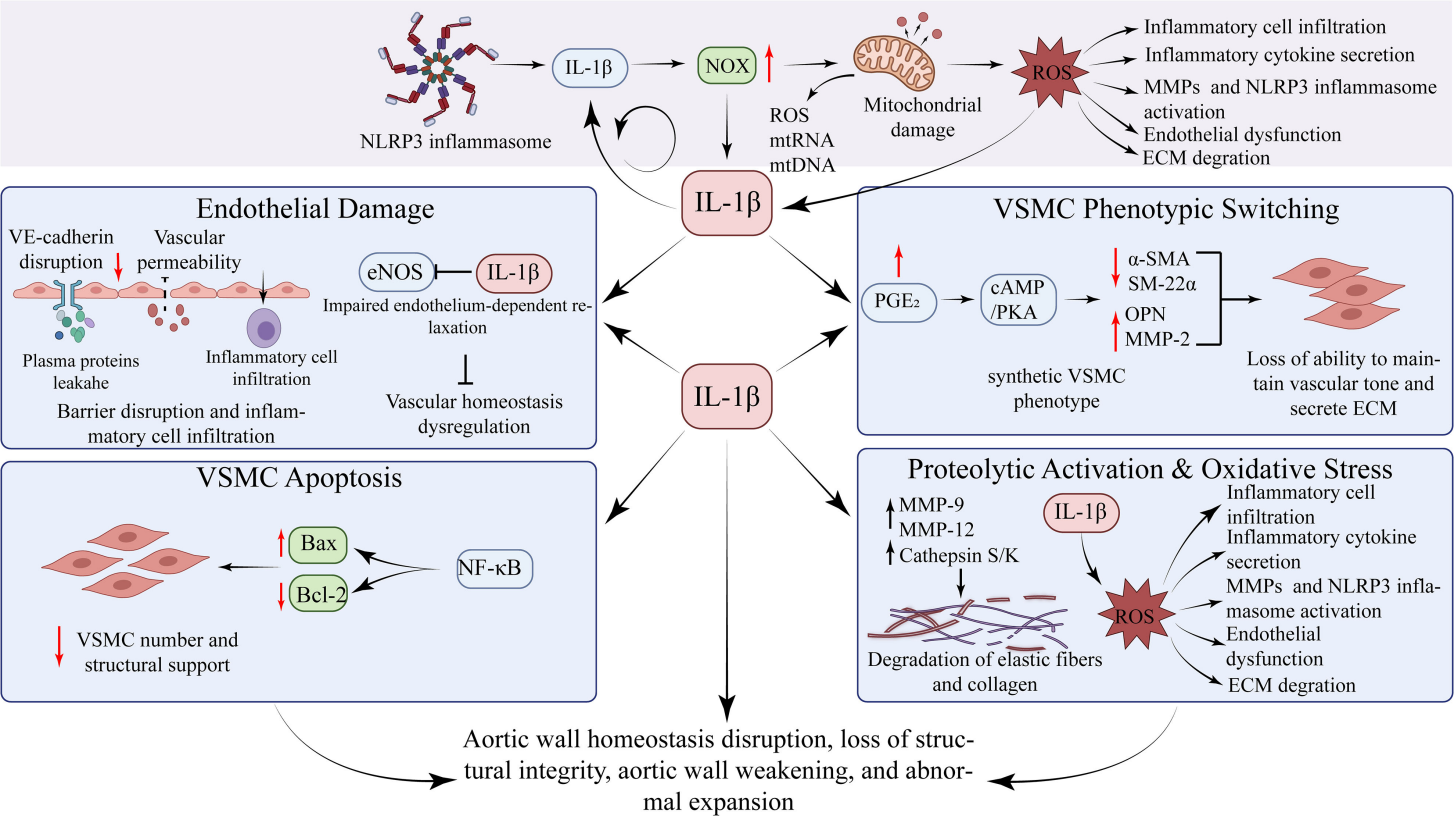
IL-1β-mediated disruption of aortic wall homeostasis. IL-1β contributes to aortic aneurysm progression by inducing endothelial dysfunction, VSMC apoptosis, VSMC phenotypic switching, oxidative stress, and proteolytic activation. These processes promote inflammatory cell infiltration, degradation of elastin and collagen, loss of vascular structural integrity, and progressive aortic wall weakening and dilation. α-SMA, alpha-smooth muscle actin; Bax, Bcl-2-associated X protein; Bcl-2, B-cell lymphoma 2; cAMP/PKA, cyclic adenosine monophosphate/protein kinase A pathway; ECM, extracellular matrix; eNOS, endothelial nitric oxide synthase; GSDMD, gasdermin D; IL, interleukin; MMPs, matrix metalloproteinases; mtDNA, mitochondrial DNA; mtRNA, mitochondrial RNA; NLRP3, NOD-like receptor family pyrin domain-containing 3; NOX, NADPH oxidase; OPN, osteopontin; PGE2, prostaglandin E2; ROS, reactive oxygen species; SM22α, smooth muscle protein 22-alpha; VE-cadherin, vascular endothelial cadherin; VSMCs, vascular smooth muscle cells.

In TAAs, the structural disruption caused by IL-1β is more commonly characterized by medial degeneration, VSMC apoptosis or phenotypic abnormalities, elastic fiber rupture, and ECM homeostasis imbalance (115). In AAA, IL-1β-associated vascular wall injury is typically dominated by an inflammation-proteolysis network centered on macrophages, neutrophils/NETs, VSMCs, MMPs, and mural thrombi (49, 116). These IL-1β-centered mechanisms and their interactions are summarized in **Figure 3**.

Under physiological conditions, VSMCs maintain vascular tone, regulate blood pressure, and support tissue perfusion through contraction and relaxation. In aortic aneurysms, however, VSMCs shift from a contractile to a synthetic phenotype. This transition impairs their ability to maintain vascular tone and regulate ECM production. This leads to a disruption in vascular wall homeostasis, providing the pathological basis for the formation of aortic aneurysms (117). The loss of structural integrity resulting from VSMC dysfunction, including apoptosis and extracellular matrix degradation, leads to aortic wall fragility and dilatation (118). VSMC apoptosis and phenotypic transition are central mechanisms in aortic aneurysm formation (69). IL-1β is a key pro-inflammatory cytokine in aortic aneurysms. It disrupts vascular wall homeostasis and structural integrity through several mechanisms, including endothelial injury, proteolytic system activation, VSMC apoptosis, and VSMC phenotypic transformation (6). Furthermore, IL-1β can significantly increase vascular permeability by disrupting intercellular adhesion molecules in endothelial cells (such as VE-cadherin), thereby promoting plasma protein leakage and the infiltration of inflammatory cells into the vascular wall (59). Concurrently, IL-1β inhibits the activity of endothelial nitric oxide synthase (eNOS), impairing endothelium-dependent vasodilation and further disrupting vascular homeostasis (119). Studies have shown that IL-1β/IL-1R1 gene knockout results in significant inhibition of apoptosis and reduced aneurysm diameter in AAA models, indicating that IL-1β is a key cytokine driving VSMC apoptosis (26).

IL-1β directly induces VSMC apoptosis by activating NF-κB and other downstream signaling pathways, upregulating pro-apoptotic proteins (e.g., Bax) and downregulating anti-apoptotic proteins (e.g., Bcl-2), thereby triggering the apoptotic cascade (120). Conversely, IL-1β activates the cAMP-PKA pathway by upregulating prostaglandin E2 (PGE2) expression. This leads to decreased expression of VSMC contractile markers (e.g., α-SMA, SM-22α) and increased expression of synthetic markers (e.g., OPN, MMP-2), resulting in a phenotypic shift from contractile to stellate/synthetic VSMCs (121). Apoptosis and phenotypic switching of VSMCs impair their ability to maintain vascular tension and secrete extracellular matrix, accelerating the disruption of aortic wall homeostasis and structural integrity (122). Furthermore, IL-1β activates the proteolytic system, promoting the expression and activity of matrix metalloproteinases (MMPs), particularly MMP-9 and MMP-12, which exhibit potent degradative effects on the extracellular matrix (123, 124). Excessive MMP activation leads to over-degradation of elastic fibers and collagen—core components maintaining vascular wall homeostasis—thereby compromising aortic wall structural integrity (125, 126). Furthermore, IL-1β induces VSMCs and macrophages to synthesize and secrete cathepsins S and K, exacerbating degradation of elastic fibers and collagen and disrupting aortic wall homeostasis and structural integrity (127). Simultaneously, IL-1β activates Nox enzymes on endothelial cells, macrophages, and VSMCs. These enzymes transfer electrons from NADPH to oxygen, generating superoxide anion (O_2_^-^) and abundant ROS (26, 128). ROS promote inflammatory cell infiltration, increase cytokine secretion, and activate MMPs (129). Furthermore, ROS accumulation causes endothelial dysfunction, disrupting vascular barrier integrity and permeability while promoting ECM degradation (130, 131). Excessive ROS directly damages cells and disrupts mitochondrial membranes. This reduces mitochondrial membrane potential and impairs the mitochondrial electron transport chain. Damaged mitochondria release additional ROS, creating a vicious cycle (132). Furthermore, ROS can act as a novel danger signal to reciprocally promote NLRP3 inflammasome activation (133). Particularly in the context of mitochondrial damage, the release of mtRNA and mtDNA can further enhance NLRP3 inflammasome activation in macrophages, thereby promoting the maturation and release of additional IL-1β (134). Consequently, a mutually reinforcing positive feedback loop is formed among ROS, IL-1β, the NLRP3 inflammasome, and mitochondria.

In the pathogenesis of aortic aneurysms, VSMCs serve as the core component maintaining vascular homeostasis. Under pathological conditions, the key proinflammatory cytokine IL-1β induces VSMCs to transition from the contractile to the synthetic phenotype and promotes their apoptosis, thereby compromising their normal function. Simultaneously, IL-1β activates proteolytic systems, induces oxidative stress, and causes endothelial injury through multiple mechanisms, synergistically promoting extracellular matrix degradation and vascular structural disruption. Ultimately, VSMC dysfunction, coupled with persistent inflammation and matrix degradation, collectively drives aortic wall fragility and abnormal dilation.

### 4.3 Interactions between IL-1β and other factors and inflammatory pathways

AAA typically manifests as an inflammation-proteolysis network comprising immune cell infiltration, neutrophil extracellular traps (NETs) and NLRP3 inflammasome activation, MMP-mediated ECM degradation, and VSMC injury. In contrast, the pathology of TAA places greater emphasis on VSMC origin/phenotype, ECM homeostasis, medial degeneration, and abnormalities in pathways such as TGF-β/SMAD, with inflammatory factors potentially contributing to local amplification (135–137).

In 2004, researchers Brinkmann et al. first discovered that neutrophils can capture and kill pathogens via DNA-protein complexes, expanding our understanding of neutrophil immune mechanisms (138). NETs are extracellular fiber networks released by neutrophils under specific stimuli. They are composed of nuclear or mitochondrial DNA decorated with multiple antimicrobial proteins. Their primary function is to capture and kill pathogens (139). NETs consist of a DNA scaffold and are loaded with neutrophil elastase (NE), myeloperoxidase (MPO), histones, and various granule proteins. Excessive formation of NETs or impaired clearance can lead to tissue damage and contribute to the onset and progression of various inflammatory and autoimmune diseases (140). Studies indicate co-localization of IL-1β and NETs in abdominal aortic aneurysm tissue. IL-1β promotes NET formation and neutrophil elastin release, compromising vascular wall structural integrity and accelerating aneurysm development (49, 141). IL-1β-induced NET formation constitutes a key early pathological process in aortic aneurysms (141). Furthermore, NETs activate NLRP3 inflammasomes in macrophages, leading to additional IL-1β release (6, 142). IL-1β knockout mice exhibit markedly reduced neutrophil infiltration, demonstrating IL-1β’s role in driving NETs-mediated aneurysm formation (143).

Moreover, IL-1β interacts with IL-6 and TGF-β within aortic aneurysms. Studies indicate that IL-1β significantly upregulates IL-6 production via the NF-κB pathway in aortic aneurysms (143). IL-6 binds to the membrane-associated IL-6 receptor (mIL-6R) on target cell surfaces, activating JAK family kinases that subsequently phosphorylate STAT3 (144). Phosphorylated STAT3 dimerizes and translocates to the nucleus, further upregulating IL-1β and TNF-α expression, forming a positive feedback loop of inflammation (26). The IL-6/STAT3 signaling pathway also promotes phenotypic transformation and inflammatory activation of VSMCs, causing them to transition from homeostatic cells that maintain vascular contractility to pathological cells involved in vascular inflammation and pathological remodeling (145, 146). Concurrently, IL-6 induces MMP-2 and MMP-9 expression and apoptosis in vascular smooth muscle cells (26, 144). Furthermore, IL-1β can also induce vascular endothelial proliferation and inflammatory cell infiltration through the JAK/STAT3 pathway, amplifying inflammation (147). At the same time, activation of STAT3 promotes the expression of matrix metalloproteinases (MMPs) such as MMP-2 and MMP-9; these MMPs degrade elastin and collagen, leading to an imbalance in ECM homeostasis, rupture of the elastic lamina, and a decrease in the mechanical strength of the aortic wall (148, 149). TGF-β is generally considered a key regulator of vascular wall homeostasis; moderate TGF-β signaling helps suppress excessive infiltration of innate immune cells and inflammatory activation, thereby exerting a protective effect on the aortic wall (150). In vascular smooth muscle cells, loss of TGF-β signaling activates the intracellular IL-1β autocrine pathway, upregulating IL-1β expression and promoting aortic aneurysm development (151). In Ang II-induced inflammatory AAAs, loss of TGF-β signaling disrupts immune homeostasis. This exacerbates monocyte/macrophage-mediated inflammation and matrix degradation, thereby promoting aneurysm progression, complications, and rupture (152). Studies indicate that in C57B/6 mouse models, TGF-β signaling activation suppresses innate immune cell infiltration and activation, exerting a protective effect in aortic aneurysms (152). However, TGF-β also plays a pathogenic role. In Marfan syndrome, excessive TGF-β signaling promotes vascular matrix remodeling and vascular smooth muscle cell dysfunction, further exacerbating aortic aneurysms (153, 154).

## 5 Therapeutic strategies targeting IL-1β in aortic aneurysms and research advances

### 5.1 Experimental evidence for targeting IL-1β in aortic aneurysm treatment

As previously discussed, IL-1β is a key pro-inflammatory cytokine in aortic aneurysm development, promoting inflammatory cell infiltration, VSMC apoptosis, and phenotypic conversion. It also upregulates MMPs, adhesion molecules, chemokines, ICAM-1, and VCAM-1 expression, playing a crucial role in aneurysm progression. Therefore, targeting IL-1β represents a potential therapeutic strategy.

Multiple experimental studies demonstrate that IL-1β expression is significantly upregulated in both cellular and animal models of aortic aneurysms (65). Inhibition of the IL-1β signaling pathway effectively reduces inflammatory cell infiltration and downregulates the expression of MMPs, ICAM-1, VCAM-1, and MCP-1 (155). It also suppresses VSMC apoptosis and phenotypic conversion (26).Studies indicate that in Ang II-infused ApoE^-/-^ mouse models and CaCl_2_-induced AAA models, IL-1β-KO or IL-1R-KO mice exhibit significantly reduced aortic aneurysm expansion rates and lesion incidence (156).

### 5.2 IL-1β receptor antagonists

#### 5.2.1 Permission to reuse and copyright

Anakinra is essentially a human endogenous IL-1 receptor antagonist produced through recombinant DNA technology, structurally highly similar to natural IL-1RA (157). As the first FDA-approved selective interleukin-1 receptor antagonist, Anakinra blocks inflammatory signaling by directly competitively inhibiting the receptor (158). Anakinra acts on IL-1β by binding with high affinity to the IL-1 receptor on the cell surface, competitively inhibiting the binding of IL-1β to IL-1R1. This prevents the formation of the IL-1R1/IL-1RAcP signaling complex and reduces IL-1β activity. As a result, key pro-inflammatory pathways such as NF-κB are suppressed, thereby interrupting the inflammatory cascade in aortic aneurysms (26). Although primarily acting as a receptor antagonist, Anakinra possesses favorable pharmacologic properties, making it one of the preferred agents for blocking the IL-1β/IL-1R1 signaling pathway. It effectively suppresses persistent inflammation in preclinical aortic aneurysm models (119).

In pathological models of aortic aneurysms, Anakinra’s therapeutic potential is strongly supported by experimental data. Studies demonstrate that compared to controls, Anakinra significantly reduces aneurysm expansion, decreases macrophage and neutrophil infiltration, and lowers VSMC apoptosis, phenotypic conversion, and extracellular matrix degradation, thereby preserving aortic wall structural integrity (159). Simultaneously, in a mouse model of abdominal aortic aneurysm induced by angiotensin II combined with a high-fat diet, mice treated with escalating doses of Anakinra exhibited a clear dose-dependent protective effect (116). Anakinra not only suppresses IL-1β expression but also indirectly influences the activation of key downstream inflammatory signaling pathways triggered by IL-1β, as well as the recruitment of inflammatory cells and the expression of inflammatory cytokines, adhesion molecules, chemokines, and various inflammatory mediators (160). These combined effects demonstrate that Anakinra delays aortic aneurysm progression by inhibiting IL-1β-driven inflammation and disrupting the vicious inflammatory cycle of aneurysms.

Currently, Anakinra treatment for aortic aneurysms remains in the early exploratory phase with limited direct clinical evidence. However, its application in other inflammation-related diseases demonstrates its potential for treating aortic aneurysms. Clinically, Anakinra is primarily used to treat inflammatory responses in rheumatoid arthritis, where its safety, efficacy, and tolerability have been validated (161). Although daily dosing may pose challenges for long-term management, the optimal therapeutic window remains to be defined (162). Concurrently, Anakinra treatment for Kawasaki disease complicated by coronary aneurysms represents a current research focus. Clinical evidence indicates Anakinra is safe and effective in stages I/IIb of aneurysmal Kawasaki disease, demonstrating efficacy in reducing inflammation and reversing aneurysms (163).

#### 5.2.2 Rilonacept

Rilonacept is a biologic agent targeting the IL-1 signaling pathway. Its mechanism of action is similar to that of Anakinra, both functioning by capturing IL-1β before it binds to the IL-1 receptor. It was approved by the FDA in 2008, becoming the first treatment for cold pyrazinic-associated periodic syndrome (CAPS) (164). To exert its potent pro-inflammatory effects, IL-1β must bind to two receptors: the interleukin-1 receptor (IL-1R1) and the accessory protein (IL-1RAcP), thereby forming a signaling-competent receptor complex (165). Rilonacept is a soluble decoy receptor composed of the extracellular domains of IL-1R1 and IL-1RAcP fused to the Fc segment of human IgG1. This structure enables it to bind IL-1α, IL-1β, and IL-1Ra with high affinity. By preventing these ligands from interacting with cellular receptors, Rilonacept effectively inhibits IL-1-mediated inflammatory signaling (26, 164). Concurrently, Rilonacept’s IgG1 Fc tail significantly prolongs its half-life in the bloodstream to several days, supporting a lower dosing frequency (166).

Unlike antagonists such as Anakinra, which compete for binding to cell membrane receptors, Rilonacept acts exclusively at extracellular sites (164). By extensively and continuously binding and “clearing” circulating IL-1β, Rilonacept fundamentally reduces the likelihood of IL-1β encountering functional receptors on target cells, thereby inhibiting the initiation of downstream inflammatory signaling (167).

Currently, clinical research on Rilonacept for aortic aneurysms remains in the early exploratory phase, lacking direct clinical application evidence. Existing studies have demonstrated Rilonacept’s efficacy in treating IL-1-mediated diseases such as CAPS, DIRA (Deficiency of IL-1 Receptor Antagonist), and RP (Recurrent Pericarditis) (168, 169). In CAPS, Rilonacept significantly reduces patients’ inflammation scores and demonstrates long-term safety (170). Additionally, Rilonacept achieves disease remission and improves quality of life in DIRA patients (171). For recurrent pericarditis, Rilonacept markedly lowers CRP levels (169).

Notably, Rilonacept achieves sustained and stable inflammation control with a once-weekly dosing regimen, highlighting its advantages in long-term management regarding compliance and convenience (171). This holds significant clinical importance for aortic aneurysms characterized by chronic inflammation.

Although direct clinical data for aortic aneurysms remain limited, Rilonacept’s long-acting mechanism and demonstrated safety and efficacy across multiple chronic inflammatory diseases provide substantial theoretical basis and clinical promise for exploring its potential role in inhibiting IL-1β inflammatory signaling and delaying disease progression in aortic aneurysms.

#### 5.2.3 Canakinumab

Canakinumab was approved by the FDA in 2009 for the treatment of Familial Cold-Induced Spontaneous Inflammatory Syndrome (FCAS) and Muckle-Wells Syndrome (MWS), with its safety and efficacy validated (172, 173). Canakinumab is a fully humanized monoclonal antibody, meaning its protein sequence is almost entirely derived from humans. This significantly reduces immunogenicity, making it suitable for long-term use (174). Canakinumab targets IL-1β by specifically recognizing and binding to it with high affinity, forming a complex that prevents its interaction with IL-1R1. This blocks the activation of key downstream signaling pathways, thereby achieving therapeutic effects against inflammation (175). Concurrently, Canakinumab does not react with IL-1α or IL-1R1. This specificity ensures targeted IL-1β inhibition for therapeutic anti-inflammatory effects while minimizing impact on other physiological or functional responses potentially involving IL-1α and IL-1R1 (176). Canakinumab’s significance in cardiovascular medicine was elevated by the CANTOS trial. This trial demonstrated that Canakinumab’s targeting of IL-1β reduces the risk of adverse cardiovascular events in patients with prior myocardial infarction and persistent inflammation (hsCRP ≥ 2 mg/L). This confirms its central role in cardiovascular diseases like atherosclerosis, a risk factor for aortic aneurysms, suggesting potential therapeutic effects on aortic aneurysm inflammation (177).

Researchers have now demonstrated in animal models that canakinumab inhibits aortic aneurysm expansion in ApoE^-/-^ mouse models (178). Additionally, studies using 01BSUR—a mouse “equivalent site” analog of canakinumab—and molecular MRI technology revealed significantly reduced inflammation in mouse aortic aneurysms following 01BSUR treatment (179).

Research on canakinumab for aortic aneurysms remains in the exploratory phase of clinical trials, with its safety and efficacy still requiring validation in clinical studies.

### 5.3 Inhibitors targeting upstream of IL-1β

Beyond directly targeting IL-1β, therapeutic approaches can indirectly reduce IL-1β expression by inhibiting upstream key signaling pathways such as the NLRP3 inflammasome. This blocks persistent inflammatory responses in aortic aneurysms, achieving therapeutic goals.

MCC950 is currently the most extensively studied selective NLRP3 inhibitor, acting upstream of the IL-1β production pathway (180). MCC950 binds to the NACHT domain of NLRP3, inhibiting its ATPase activity and oligomerization process, thereby affecting the assembly and activation of the NLRP3 multiprotein complex (181). Since NLRP3 is a key regulatory pathway for IL-1β maturation and release, MCC950 effectively and indirectly suppresses IL-1β expression, exerting anti-inflammatory effects against aortic aneurysms.

Research indicates that in mouse models of abdominal aortic aneurysm induced by high-fat, high-cholesterol diet combined with angiotensin II infusion or CaCl_2_ administration, treatment with MCC950 significantly reduced aortic aneurysm diameter expansion by 35% and markedly slowed the rate of expansion (182). Further mechanistic analysis revealed that MCC950 intervention significantly decreased levels of key pro-inflammatory cytokines such as IL-1β, IL-6, and TNF-α in mouse serum, while also reducing macrophage infiltration in the vascular wall (179). Concurrently, studies revealed that NLRP3^-/-^, Casp1^-/-^, and IL-1β^-/-^ knockout mice exhibited marked resistance to aortic aneurysm development under identical induction conditions (182). MCC950 also inhibited VSMC apoptosis and phenotypic conversion, reduced ECM degradation, and maintained aortic wall structural integrity and homeostasis (182, 183).

These findings collectively demonstrate that MCC950 indirectly suppresses the expression of multiple key pro-inflammatory cytokines, including IL-1β, by inhibiting NLRP3, thereby alleviating aortic aneurysm inflammation. Further exploration is needed for the clinical application of MCC950 in aortic aneurysms.

OLT1177 is another highly selective NLRP3 inflammasome inhibitor that blocks NLRP3 inflammasome assembly and activation by inhibiting its ATPase activity (184). Studies indicate that OLT1177 significantly reduces the release of IL-1β and IL-18, thereby alleviating inflammation (185). Additionally, OLT1177 improves vascular endothelial cell function and indirectly inhibits oxidative stress by suppressing NLRP3 activity (184, 186). Currently, OLT1177 remains in clinical development for aortic aneurysm treatment. However, studies have demonstrated its favorable safety and tolerability in acute gout attacks and chronic systolic heart failure, suggesting its therapeutic potential for aortic aneurysms warrants further exploration (185).

Furthermore, TEPP-46, as a metabolic modulator, represents an innovative strategy to intervene in inflammation from an energy metabolism perspective. It can inhibit NLRP3 inflammasome-mediated IL-1β secretion by activating pyruvate kinase M2 (PKM2) (187). Further studies indicate that TEPP-46 mitigates aortic aneurysm progression in animal models of thoracic aortic aneurysms and dissections by inhibiting the NLRP3-IL-1β pathway (187). This finding reveals an intrinsic relationship between cellular metabolic reprogramming and inflammasome activation, offering a novel perspective for metabolically-targeted interventions in chronic inflammatory diseases like aortic aneurysms.

Currently, most NLRP3 inhibitors for aortic aneurysms remain in preclinical or early clinical stages. However, clinical trial data from related chronic inflammatory diseases like atherosclerosis, gout, and diabetes provide mechanistic efficacy and safety references for aortic aneurysm treatment (180). Concurrently, addressing long-term vascular inflammation in chronic aortic aneurysms requires consideration of NLRP3 inhibitor safety during prolonged use, precision targeting, and synergistic effects with other medications.

IL-1β receptor antagonists and related IL-1β-targeted biologics primarily inhibit downstream inflammatory signaling by blocking the binding of IL-1β to its receptor, trapping free IL-1β, or specifically neutralizing IL-1β. To facilitate comparison of the target sites, mechanisms of action, and research progress of different drugs, the main IL-1β-targeted drugs mentioned above are summarized below (**Table 1**).

**Table 1:** Summary of drugs for the treatment of aortic aneurysms targeting the IL-1β/IL-1 receptor signaling pathway.

Medications	Drug types	Mechanism of action	Evidence from studies on aortic aneurysms	Current status of clinical research	Key features
Anakinra	IL-1 receptor antagonist	Competitively binds to IL-1R, blocking IL-1β/IL-1R1 signaling	The model demonstrated reduced vasodilation, inflammation, and matrix degradation; in the Ang II-induced model combined with a high-fat diet, it exhibited a dose-dependent protective effect.	Early	FDA-approved; good safety and tolerability
Rilonacept	Soluble IL-1 decoy receptor	Binds with high affinity to IL-1α, IL-1β, and IL-1Ra, preventing them from binding to their cellular receptors	Direct clinical evidence is limited, and research in the field of aortic aneurysms remains in its early stages	Lack of direct clinical evidence; has been applied or validated in conditions such as CAPS, DIRA, and recurrent pericarditis	Long-lasting and well-tolerated
Canakinumab	Fully humanized anti-IL-1β monoclonal antibody	Specifically recognizes and binds to IL-1β with high affinity, preventing it from binding to IL-1R1	Inhibition of dilation in the ApoE^-/-^ mouse model; MRI revealed reduced inflammation in the aortic aneurysm	Exploratory phase of clinical trials	Highly specific, low immunogenicity, and suitable for long-term use
MCC950	Selective NLRP3 inflammasome inhibitors	Inhibits ATPase activity and oligomerization, thereby preventing the assembly and activation of the NLRP3 inflammasome	Inhibits vasodilation and reduces inflammatory damage in Ang II or CaCl_2_ models	Further clinical research is needed	Classic NLRP3 inhibitors
OLT1177	Highly selective NLRP3 inflammasome inhibitor	Inhibiting the ATPase activity of the NLRP3 inflammasome	Limited evidence is available, and it is still in the clinical development stage	It has demonstrated good safety and tolerability	High selectivity, good safety profile
TEPP-46	Metabolic modulator; PKM2 activator	Activate PKM2 while inhibiting the NLRP3 inflammasome	Alleviating disease progression by inhibiting the NLRP3-IL-1β pathway in TAAs and *in vivo* animal models	Preclinical research phase	Intervening in inflammation from an energy metabolism perspective

## 6 Clinical research implications

The CANTOS trial represents a landmark clinical study in cardiovascular medicine, providing the first evidence that specific treatment of inflammation itself can reduce cardiovascular event risk (188). In the CANTOS trial, based on the hypothesis that canakinumab could reduce the risk of major adverse cardiovascular events in patients with existing atherosclerotic cardiovascular disease, 10,061 patients with a history of myocardial infarction and persistently elevated high-sensitivity C-reactive protein (hsCRP) levels (≥2 mg/L) were randomly assigned to receive either canakinumab or placebo. Results demonstrated that compared to placebo, canakinumab reduced hsCRP levels and the risk of major adverse cardiovascular events (189). The CANTOS trial confirmed that targeting the IL-1β inflammatory pathway effectively suppresses systemic and vascular wall inflammation, thereby significantly lowering the risk of major adverse cardiovascular events (189). Although this trial did not directly target aortic aneurysm treatment itself, its findings offer a novel perspective on understanding the etiology and pathogenesis of atherosclerotic diseases, including aortic diseases. It provides theoretical basis and translational directions for exploring IL-1β pathway targeting to stabilize aortic structure and delay aneurysm progression.

This reveals that the treatment paradigm for cardiovascular diseases dominated by chronic inflammation is shifting from initial imaging and surgical interventions toward a new comprehensive treatment model targeting inflammation.

The etiology of aortic aneurysms is complex, involving multiple pathological types such as atherosclerosis, genetic syndromes, and autoimmune-related diseases (190). Personalized treatment for aortic aneurysms will not only be based on aneurysm diameter and growth rate but may also incorporate the patient’s inflammatory status. Tailored treatment plans developed in conjunction with inflammatory responses will become key perioperative care points, addressing the high heterogeneity of patient populations. Only through precise stratification strategies can therapeutic benefits be maximized while avoiding the risks and costs associated with ineffective treatments.

## 7 Therapeutic strategies targeting IL-1β in aortic aneurysms and research advances

### 7.1 Current challenges

The etiology of aortic aneurysms is complex, with patient populations exhibiting high heterogeneity in genetic background, tissue architecture, cellular composition, hemodynamic characteristics, and clinical manifestations (191). Not all aortic aneurysm subtypes rely on IL-1β axis activation for progression. For instance, familial non-syndromic thoracic aortic aneurysms and dissections (TAAD) primarily stem from distinct familial genetic backgrounds involving mutations in smooth muscle contraction genes such as actin and myosin. In contrast, hereditary syndromes like Marfan syndrome primarily result from connective tissue structural abnormalities caused by mutations in extracellular matrix-related genes (e.g., fibronectin, TGF-β receptors) (192, 193). Furthermore, although IL-1β is highly expressed in aortic aneurysms, it may also be elevated in other inflammatory diseases, resulting in low specificity (26). Therefore, administering IL-1β inhibitors to all aortic aneurysm patients would not only yield limited efficacy but could also cause unnecessary side effects. .

Identifying truly inflammation-driven aortic aneurysm subtypes using reliable biomarkers is critically important. Potential biomarkers include serum markers such as hsCRP and IL-6, as well as imaging biomarkers—particularly positron emission tomography (PET) combined with specific tracers like ^18^F-FDG and ^18^F-NaF) to qualitatively and quantitatively assess inflammatory activity in aortic aneurysms (194–196). Biomarkers currently under investigation or with potential for use in inflammation stratification are listed in **Table 2**. Biomarker-based precision stratification holds promise for enabling progressive, personalized treatment of aortic aneurysm patients, thereby reducing the incidence of aortic aneurysms.

**Table 2:** Biomarkers currently under investigation or expected to be used for inflammation stratification. .

Marker types	Signature markers	Current Research focus	Conversion potential	Main limitations	References
Traditional inflammatory markers	hsCRP, CRP, IL-6	Assess the inflammatory burden and determine the state of inflammatory activity	Intermediate	Low specificity	(197, 198)
Proteins associated with matrix degradation	MMP-9	Assessment of ECM degradation, elastic fiber disruption, and tissue remodeling activity	Higher	A single MMP marker is insufficient to reflect the overall pathological process	(199)
Circulating Protein Profile	MMP-9+IL-6+hsCRP;Combined D-dimer, OPN, OPG, etc.	Identify the inflammation-active type and the matrix degradation-active type	Higher	Lack of a standardized panel of tests and clinical cutoff values	(200–202)
Inflammasome-associated markers	IL-1β, IL-18, caspase-1, NLRP3, ASC	Assessment of NLRP3 inflammasome activation and IL-1β release	relatively high but still in the research phase	Lack of stability and standardization in peripheral blood testing	(12, 203)
microRNA biomarkers	miR-21, miR-29, miR-146a, miR-155	VSMC phenotypic conversion, macrophage activation, and ECM remodeling	Above average	Lack of consistency among different study results	(204–207)
^18^F-FDG PET/CT imaging markers	SUVmax, TBRblood, TBRliver	Determine the optimal cutoff value for diagnosing large-vessel vasculitis (LVV), assess its correlation with disease activity (CRP), and distinguish between active vasculitis and atherosclerosis (AS)	Higher	The cutoff values have not yet been standardized, making it impossible to assess the risk of rupture.	(208–210)
18F-NaF PET/CT imaging markers	NaF uptake values, microcalcification signals in the aortic wall	Assessment of microcalcifications, active remodeling of lesions, and aortic wall instability	Above average	has not yet become a standard risk assessment tool	(211, 212)
Multi-omics integrated biomarkers	Omics proteomics, transcriptomics, single-cell RNA sequencing (scRNA-seq), spatial transcriptomics	Identification of inflammatory cell subsets, candidate biomarkers, and spatial distribution of lesions	High research potential	High costs and a relatively long time to clinical translation	(213–216)

The aforementioned biomarkers are all candidates currently under investigation or with potential translational value for the stratification of aortic aneurysm inflammation, the prediction of disease progression, or the identification of IL-1β/NLRP3 pathway activity.

IL-1β exhibits dual roles in physiological and pathological contexts, functioning both as a defensive molecule and a major destructive factor. During infection, IL-1β coordinates local and systemic inflammatory defense responses through autocrine, paracrine, and endocrine mechanisms (25). Prolonged suppression of IL-1β activity may compromise the body’s anti-infective capacity and increase infection risk, particularly when combined with other immunosuppressants (217). Given that aortic aneurysm patients are predominantly elderly and often have comorbid chronic diseases, particular caution regarding infection risk is warranted when employing inhibitors (218). Therefore, a careful balance must be struck between the benefits and potential risks of inhibitor therapy for aortic aneurysms. Alternatively, exploring the lowest effective dose of IL-1β inhibitors, optimizing dosing regimens, or developing novel *in vivo* delivery methods could achieve therapeutic efficacy while minimizing infection risk.

Although preclinical evidence for IL-1β-targeted therapy in aortic aneurysms is robust, significant challenges remain in clinical translation. Monotherapy with IL-1β inhibitors alone cannot meet clinical needs and requires combination with other therapeutic targets (219, 220). Future treatment of aortic aneurysms will progressively evolve toward precision medicine-guided personalized therapy while developing additional combination treatment strategies.

### 7.2 Future research directions

The etiology of aortic aneurysms is complex, involving multiple pathological factors such as inflammation, abnormal protease activity, VSMC apoptosis, and matrix degradation. IL-1β may play a dominant role only in specific disease stages, meaning single-target interventions cannot fully halt disease progression and require combination with other drugs. Combining IL-1β inhibitors with other anti-inflammatory drugs or agents targeting different inflammatory pathways may enhance synergistic effects. Potential combination therapies include: ① Renin-angiotensin system inhibitors, such as losartan (angiotensin II receptor blocker, ARB) and captopril (angiotensin-converting enzyme inhibitor, ACEI), which suppress inflammatory responses while downregulating MMP activity to exert protective effects (221, 222). ② Antiplatelet agents: e.g., aspirin. Retrospective cohort studies have associated aspirin or clopidogrel with reduced aortic aneurysm mortality (77). ③ Statins, which inhibit the NF-κB signaling pathway, reduce MMP activity, and modulate cytokines, thereby suppressing inflammatory responses in aortic aneurysms (223). ④ Drugs targeting other pathways, such as those targeting IL-6 or TNF-α, offer additional possibilities for multi-pathway synergistic intervention. Among these, the combination of IL-1β inhibitors and RAS inhibitors may be the most promising. The Ang-AT1R axis drives hemodynamic stress, oxidative stress, macrophage recruitment, MMP activation, and aortic wall remodeling (224–226). Meanwhile, IL-1β occupies a key node in the inflammatory amplification loop, driving macrophage and neutrophil recruitment, VSMC injury, and proteolytic reactions. The combination of these two agents could theoretically produce a synergistic effect involving “upstream trigger inhibition + downstream inflammatory amplification blockade.” However, there is currently a lack of systematic studies directly comparing the efficacy, dose-response relationships, optimal intervention strategies, and long-term safety of the “IL-1β inhibitor + RAS inhibitor” combination therapy versus monotherapy in aortic aneurysms.

Furthermore, aortic aneurysms represent a localized inflammatory destructive disease of the vascular wall. However, current targeted inhibitors administered via subcutaneous or intravenous injection result in systemic drug distribution, meaning only a fraction reaches the diseased aortic wall (227). Achieving locally effective concentrations requires higher doses, which not only increases treatment costs but also amplifies the risk of potential systemic side effects. Therefore, there is a need to develop highly effective targeted inhibitors and explore efficient delivery methods to achieve specific targeting and release of drugs within aortic aneurysms. This approach maximizes local therapeutic efficacy while minimizing the risk of systemic side effects. Currently, intratumoral injection and surgically assisted local administration may maximize localized efficacy by physically placing or attaching targeted drugs directly to the lesion site, enabling precise, sustained local treatment (228, 229).

Given the complexity of aortic aneurysms and the high heterogeneity of patient populations, future treatment will trend toward precision personalized therapy and multidisciplinary integration. Inflammation-guided personalized therapy requires accurate identification of patients who are truly dependent on the IL-1β signaling pathway. Future studies should integrate multidimensional data, including imaging biomarkers, genomics, transcriptomics, proteomics, and single-cell omics. This approach may enable a more refined inflammatory classification of aortic aneurysms. In particular, single-cell RNA sequencing, spatial transcriptomics, and multi-omics integration analysis can be used to identify IL-1β-active cell populations with high expression of inflammatory genes such as IL-1β, NLRP3, and caspase-1, and to elucidate the ligand-receptor communication networks among macrophages, neutrophils, endothelial cells, and VSMCs (230, 231). Spatial transcriptomics can address the lack of tissue localization information in scRNA-seq, localizing inflammatory cell subsets and inflammation/tissue remodeling-related signals to specific pathological regions such as media damage, disruption of the elastic lamina, intimal inflammatory infiltration, and ECM remodeling (232). When further integrated with clinical phenotype and treatment response data, these techniques hold promise for identifying IL-1β-dependent patient subgroups, discovering targets for combination therapy, and monitoring changes in inflammatory cell composition and vascular wall repair responses following treatment with IL-1β/NLRP3 inhibitors, thereby enabling precision-targeted interventions for aortic aneurysms.

## 8 Conclusion

Given the rising incidence of aortic aneurysms and the clinical challenges they present, current evidence indicates that IL-1β serves as a core driver of the inflammatory cascade in aortic aneurysms. Preclinical studies demonstrate that targeting IL-1β and its upstream signaling pathways effectively delays aneurysm progression. This offers a highly promising breakthrough for addressing the current therapeutic challenges in aortic aneurysm management. Although the clinical translation of drugs targeting IL-1β and its upstream signaling pathways still faces challenges such as patient heterogeneity and long-term safety, in-depth exploration of IL-1β-targeted therapy holds significant practical importance for developing precision drug treatment strategies to delay aortic aneurysm progression and reduce the risk of aneurysm rupture.
